# Flexible and Multifunctional Composites with Highly Strain Sensing and Impact Resistance Properties

**DOI:** 10.3390/polym16111544

**Published:** 2024-05-30

**Authors:** Shu Wang, Jianyu Pu, Shuquan Xu, Yuanhao Tian, Qian Shu, Rui Zou, Tonghua Zhang

**Affiliations:** 1 State Key Laboratory of Resource Insects, College of Sericulture, Textile and Biomass Sciences, Southwest University, Chongqing 400715, China; wangshu@swu.edu.cn (S.W.); p836149527@email.swu.edu.cn (J.P.); 2 College of Aerospace Engineering, Chongqing University, 174 Shazheng St., Shapingba District, Chongqing 400044, China; 3 Southwest Technology and Engineering Research Institute, Chongqing 400039, China; yuanhao_tian@foxmail.com (Y.T.); 17723898496@163.com (Q.S.); 4 School of Materials Science and Engineering, Chongqing Jiaotong University, Chongqing 400074, China; ruizou@cqjtu.edu.cn

**Keywords:** graphene, 3D fabric, mechanical properties, piezoresistive performance, structure sensor

## Abstract

The development of smart protective clothing will help detect injuries from contact sports, traffic collisions, and other accidents. The combination of ecoflex, spacer fabric, and graphene-based aerogel provides a multifunctional composite. It shows a strain sensitivity of 17.71 at the strain range of 40~55%, a pressure sensitivity of 0.125 kPa^−1^ at the pressure range of 0~15 kPa, and a temperature sensitivity of −0.648 °C^−1^. After 50 impact tests, its protection coefficient only dropped from 60% to 55%. Additionally, it shows thermal insulation properties. The compression and impact process results of finite element numerical simulation analysis are in good agreement with the experimental results. The ecoflex/aerogel/spacer fabric sensor exhibits a simple structure, large pressure strain, high sensitivity, flexibility, and ease of fabrication, making it a candidate for smart protective clothing resistant to impact loads.

## 1. Introduction

Smart textiles have broad applications in military, aerospace, and medical fields. In the early stage, smart textiles [1,2] were fabricated by mechanically embedded sensors (including capacitive, resistive, and optical sensors [1]) in fabrics. With the increasing requirements on the flexibility and wearability of electronic devices, modified fabrics as direct smart fabric sensors [3,4] have attracted more research attention. They can detect strain, pressure, temperature, humidity, human body movements [5], angular displacement [6], and other signals, which have important application prospects in various fields [7] such as military affairs, security, medical treatment, sports, and energy storage [8,9]. Due to the limitations of technology and materials, the compression resistance and impact resistance of traditional smart fabric flexible sensors in extreme environments are very limited. It is very important for soldiers to resist the impact of bullets and shell fragments. Being able to monitor the health status of soldiers at any time is of great significance to the battlefield situation [10]. Therefore, more and more attention has been paid to anti-impact protective equipment with specific sensing characteristics, because it can reduce and dynamically monitor the external impact force suffered by soldiers, so that they can be treated in a timely manner. Therefore, an investigation of smart textile-based materials with sensing and impact protection is essential. 

Wearable smart devices are developing rapidly as a new generation of electronic products. Compared with the traditional metal-foil strain gauges with poor stretchability and high hardness, piezoresistive strain sensors based on conductive polymer composites (CPCs) exhibit better flexibility and conformability; hence, CPCs are more suitable for application in wearable electronics [11]. CPCs are fabricated by integrating conductive nanomaterials into elastic polymer substrates through mixing and coating processes to establish stretchable conductive networks [12]. The working principle of CPC-based flexible strain sensors is that the deformation of the elastic polymer substrate causes destruction and reconstruction of the conductive network during applied loading and unloading, resulting in a change in electrical resistance [13]. In recent years, various CPC-based sensors have been developed, including multifunctional hydrogel sensing materials [14], epoxy-based conductive nanocomposites [15], PDMS-based composite strain sensors [16], electrically conductive fabrics [17] and graphene–silk fabric strain sensors [18]. These sensors are based on a two-dimensional level, and usually cannot be used for impact protection at the same time. Three-dimensional fabric has excellent compression properties and can be used as the substrate of textile-based flexible sensors. In recent years, many sensors and numerical models based on 3D fabrics have been developed. For example, a flexible pressure sensor based on hierarchical three-dimensional and porous reduced graphene oxide (rGO) fiber fabrics was developed [19], while Hu et al. [20] proposed a comprehensive multi-scale three-dimensional (3D) resistor network numerical model. Although some progress has been made in the research of 3D smart fabrics, several critical issues have yet to be addressed; for example, limited functionality, complex manufacturing processes, and a lack of systematic research on simultaneous sensing and impact protection.

A large amount of research has been carried out in the fabric sensing field, in particular with various options for carbon materials such as carbon nanotubes [21], CB [22], and silver nanowire [23]. However, graphene, due to its unique physical and chemical properties as a two-dimensional nanomaterial, has a wide range of applications in the field of structural and functional nanocomposites [24,25,26,27,28,29]. Spacer fabrics and ecoflex have good compression and impact resistance properties. Herein, we combined 3D spacer fabric, graphene-based aerogel, and ecoflex to fabricate a multifunctional composite. The compression, impact, and temperature sensing behaviors of composite were analyzed through experiments. We successfully demonstrated a strain/temperature sensor based on the 3D spacer fabric composite that is thermally insulating and provides impact protection. It has a strain sensitivity of 17.71 (strain is 40–55%), a temperature sensitivity of −0.648 °C^−1^, and a protection factor of 55% (even after 50 cycles). Therefore, it has broad prospects in the application of sports protection, individual combat, and health monitoring.

## 2. Material and Methods

### 2.1. Materials

Spacer fabric (height: 7 mm) was purchased from Suzhou Zhenghan Weaving Co., Ltd. (Suzhou, China). Ecoflex was purchased from Shanghai Zhixin Technology Co., Ltd. (Shanghai, China). Graphene oxide (GO, 5 mg/mL) was purchased from Suzhou Carbonfeng Technology (Suzhou, China). Ammonia (NH_3_·H_2_O, GR, 25–28%) was purchased from Shanghai Aladdin Biochemical Technology Co., Ltd. (Shanghai, China). Ultrapure water from a molecular system was used for all the experiments. All chemicals were analytic grade reagents, and used without further purification.

### 2.2. Synthesis of the Ecoflex/Aerogel/Spacer Fabric Composites

**Figure 1:**
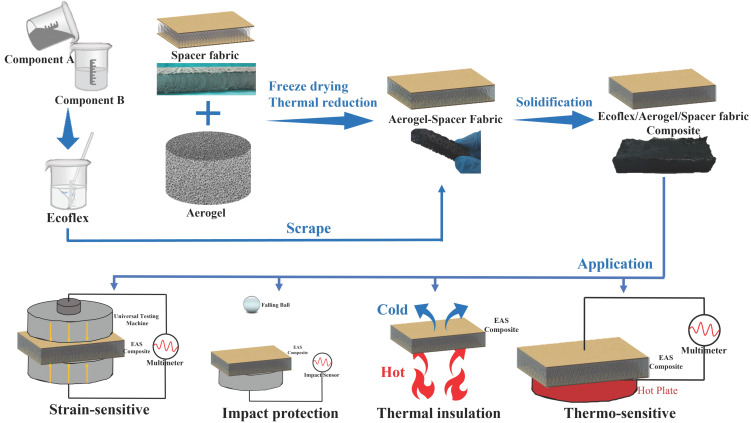
Fabrication, performance, and applications of the ecoflex/aerogel/spacer fabric composite.

Figure 1 shows the simple preparation process and application of the ecoflex/aerogel/spacer fabric composite. The GO solution was ultrasonicated in a water bath for 1 h. A certain amount of ammonia was added to adjust the pH of the GO solution to alkaline and then the water bath ultrasonic was continued for 1 h. The same amount of silk fibroin as GO was added and sonicated for 1 h. The spacer fabrics were put into molds, then the alkaline GO solution was poured into the molds. The molds were then freeze dried in a freeze dryer at −60 °C for 48 h. After being vacuum-dried in an oven at 180 °C for 6 h, the hybridized graphene-based aerogel/spacer fabric composite was created.

Subsequently, the ecoflex was obtained by mixing component A and B in the ratio of 1:1 by mass. After stirred and defoamed, the mixed ecoflex was poured on the graphene-based aerogel/spacer fabrics in a mold, and scraped repeatedly on both sides to make sure the ecoflex entered the graphene-based aerogel/spacer fabrics totally. Finally, the molds were placed in the oven for curing to obtain the ecoflex/aerogel/spacer fabric composite.

### 2.3. Preparation of the Ecoflex/Aerogel/Spacer Fabric Sensor

To prepare the ecoflex/aerogel/spacer fabric sensor, the graphene-based aerogel/spacer fabric composite was coated with conductive silver adhesive on the upper and lower surfaces, then affixed with aluminum foil electrodes which were subsequently dried in an oven at 60 °C for 3 h. After that, the ecoflex was added for brushing, so as to allow the ecoflex to enter into the graphene-based aerogel/spacer fabric composite sufficiently, then placed in the oven for curing.

### 2.4. Instruments

The materials were freeze dried using a freeze dryer (FD-1A-50+, Beijing Boyikang Instrument Co., Ltd. (Beijing, China)). The materials were sonicated using an ultrasonic cleaner (KQ2200V, Kunshan Ultrasonic Instrument Co., Ltd. (Kunshan, China)). The morphological analysis of the composite was performed using a scanning electron microscope (SEM, JSM-5610, Feina, The Netherlands) with a voltage of 10 kV. An Electromechanical Universal Testing Machine (MTS Systems Co., Ltd. (Shanghai, China)) was used to measure the mechanical properties. The compression speed was 0.1 mm/s, 2 mm/s, the strain was 5%, the ambient temperature was 20 °C, and the humidity was 65%. The impact test of composite was performed on a homemade ball impact tester. Thermal insulation performance was measured using an infrared thermal imager (H10S, HIKMICRO). All sensing performances were recorded in real time using a digital multimeter (34465A, KEYSIGHT).

## 3. Results and Discussion

### 3.1. Characterizations of the Ecoflex/Aerogel/Spacer Fabric Composites

**Figure 2:**
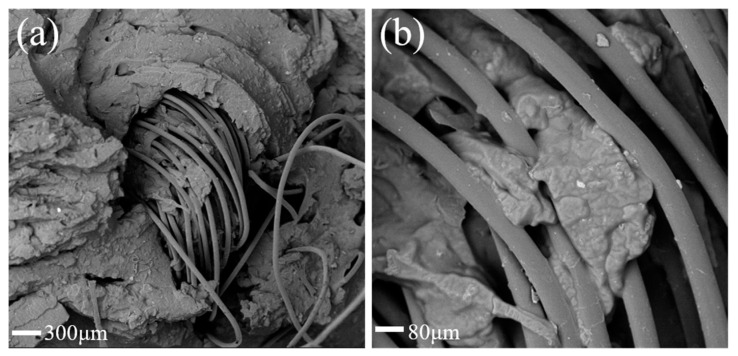
SEM images of the ecoflex/aerogel/spacer fabric composite at different magnifications.

The microstructure of the ecoflex/aerogel/space fabric composite was investigated by scanning electron microscopy (SEM). As shown in Figure 2, the ecoflex/aerogel/spacer fabric composite was filled with ecoflex and spacer filaments. This indicates that the ecoflex and the spacer fabric were equally bonded. The addition of the ecoflex stabilizes the structure of the composite, enhancing its impact resistance and strengthening the connection between graphene-based aerogel and spacer fabric. Meanwhile, the ecoflex did not infiltrate into the interior of the yarn, and the spacer filament retained its unique internal structure, indicating that the ecoflex/aerogel/spacer fabric composite still exhibited better impact-resistant performance.

### 3.2. Static Mechano-Electrical Performance of the Ecoflex/Aerogel/Spacer Fabric Sensor

**Figure 3:**
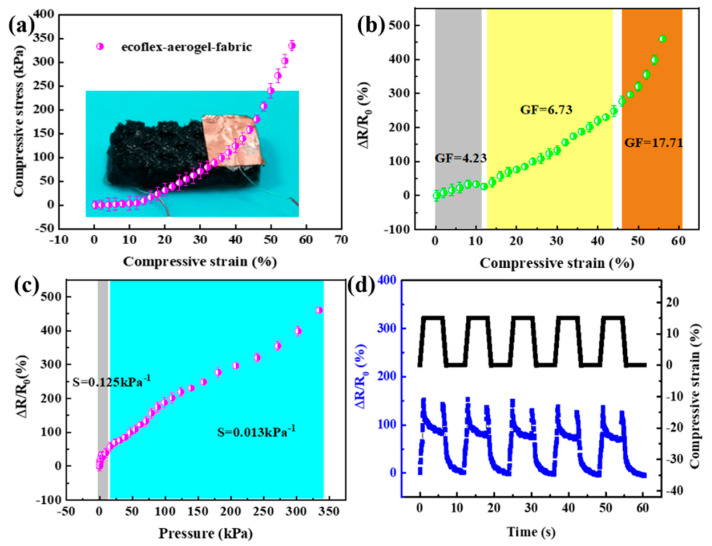
(**a**) The stress–strain curves of the ecoflex/aerogel/spacer fabric composite and the photo of composite sensor. (**b**) Relative changes of the resistance with compressive strains of the composite sensor. (**c**) Relative changes of the resistance with pressure of the composite sensor (compressive rate 0.1 mm s^−1^). (**d**) Changes of the resistance and displacement of ecoflex/aerogel/spacer fabric sensor with time delay in 15.6% strain under unloading–loading.

The spacer fabric and ecoflex endow the composite sensor with excellent compression properties, and the porous structure of the graphene-based aerogel promotes the sensitivity of the piezoresistive response. Quasi-static compressive loading tests were carried out to evaluate the strain sensing capability of the as-designed composite sensor. The stress of the composite was up to 335 kPa at a strain of 55%, as shown in Figure 3a. The relative change in resistance (ΔR/R (%), where R is the initial resistance and ΔR is the change of resistance), increased monotonically with the applied strain, as shown in Figure 3b. Generally, the sensitivity of a strain sensor is calculated by the gauge factor (GF = ΔR/R (%)/Δε), and the sensitivity of a pressure sensor is determined by S (S = ΔR/R (%)/ΔP, where ΔP is the change of pressure). As shown in Figure 3b, the GF reached 4.23 at strains of 0–10%, 6.73 at strains of 10–40%, and 17.71 at strains of 40–55%. The S reached 0.125 kPa^−1^ at pressures of 0–15 kPa and 0.013 kPa^−1^ at pressures of 15–335 kPa (Figure 3c). The behavior of the composite sensor during loading–unloading (0–15.6% compressive strain; holding time 5 s) is shown in Figure 3d. It can be seen that the ΔR/R_0_ response is consistent with the change trend of the compressive strain, indicating that the sensing performance of the three-phase composite strain sensor has been greatly improved after compounding with ecoflex, and the composite has excellent compressive sensing performance compared with the graphene-based aerogel/spacer fabric sensor [30].

Generally, the sensing performance of piezoresistive strain or stress sensors is related to the conductive path and contact resistance of conductive materials. The structural change of graphene-based aerogel in ecoflex/aerogel/spacer fabric composites is very important for its response as a piezoresistive sensor. Under compression load, the pore structure of graphene-based aerogel is destroyed, making its conductive path become discontinuous. With the increase of compression strain, the discontinuity further increases, resulting in the disconnection of conductive path, which exhibits that the resistance of the material increases, and the conductivity becomes poor. When the load is removed, the graphene-based aerogel deforms and recovers in the ideal state, and the discontinuous conductive network structure also disappears, making the resistance of the composite reduce and return to the original state [31]. During the period of maintaining constant strain, a significant decrease of ΔR/R was observed due to stress relaxation. This may due to internal stress relaxation, ΔR/R did not return to zero after each cycle in the piezoresistive sensor using PDMS as the elastomer [32,33].

**Figure 4:**
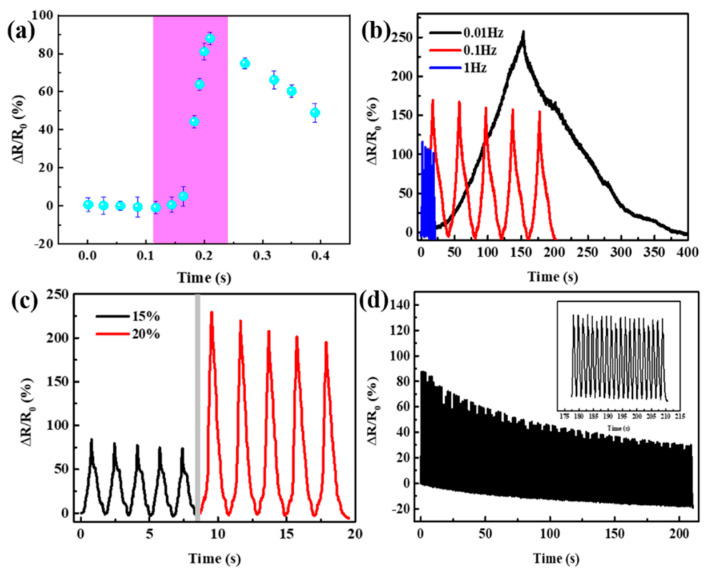
(**a**) Responses and relaxation property of ecoflex/aerogel/spacer fabric sensor. (**b**) Changes in resistance of composite sensor in different frequencies (compressive rate 2 mm s^−1^). (**c**) Multiple cycles of changes in resistance with different applied compressive strains (compressive rate 2 mm s^−1^). (**d**) Cyclic stability test results (Compressive rate 0.1 mm s^−1^, strain of 5%).

As shown in Figure 4a, the response time of the composite sensor was 92 ms. The resistance responses under compressive loading–unloading cycles with different applied strains are presented in Figure 4b,c. Clearly, the variation of ΔR/R (%) synchronized with the change of the applied strains during the loading–unloading cycles. As can be seen in Figure 4b, ΔR/R_0_ was different from graphene-based aerogel/spacer fabric sensors [30] at frequencies of 0.01 Hz, 0.1 Hz, and 1 Hz. The peak values of R_0_ were 257%, 168%, and 111%, respectively. It can be seen that the amplitude of ΔR/R_0_ decreases significantly with the increase of frequency, which is due to a certain response caused by the hysteresis after packaged by ecoflex. As shown in Figure 4c, at 15% and 20% compressive strain, the ΔR/R_0_ values were 84% and 228%, respectively. This result is superior to most other sensor [19,32,33,34]. The durability of composite sensors is another key issue for their practical application. As shown in Figure 4d, the ΔR/R (%) value of the composite sensor remained approximately constant over 120 loading–unloading cycles at a compression rate of 0.1 mm s^−1^ and a strain of 5%. This favorable durability and stability can be attributed to the unique porous structure of the hybrid graphene-based aerogel, better flexibility of the ecoflex matrix, and better compressibility of the spacer fabric.

In summary, the sensing performance of the sensors in the literature is listed in Table 1. Compared with other sensors, the ecoflex/aerogel/spacer fabric sensor’s sensitivity and working range are larger than most sensors, and are especially suitable for the detection of large stress.

### 3.3. The Dynamic Mechano-Electrical Properties and Safeguarding Performance of the Ecoflex/Aerogel/Spacer Fabric Sensor under Impact

**Figure 5:**
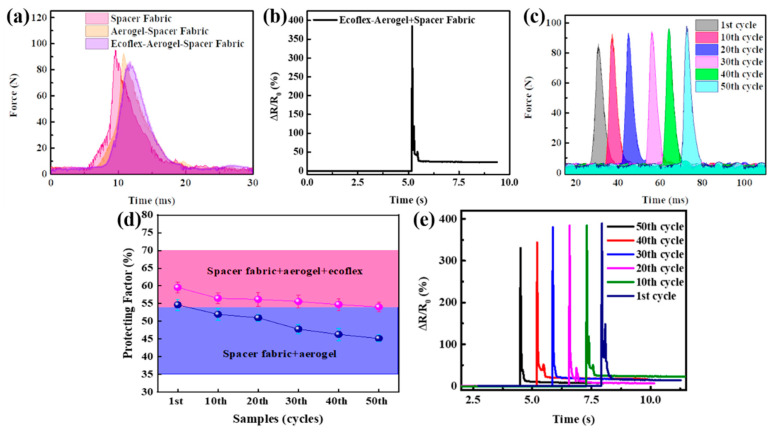
(**a**) Dynamic impact resistance tests of different samples including spacer fabric, graphene-based aerogel/spacer fabric, and ecoflex/aerogel/spacer fabric. (**b**) Stimulus-responsive sensing performance of composite under impact test. (**c**) Dynamic impact resistance tests of ecoflex/aerogel/spacer fabric. (**d**) Protecting factors for samples in cyclic impacts tests. (**e**) Stimulus-responsive sensing performance of composite under repeated impact stimuli.

Dynamic impact resistance tests were conducted to further assess the protective and sensing performance of the composite using a spherical impactor with a diameter of 2.5 cm. The impactor weighing 115 g was dropped from a height of 25 cm. As shown in Figure 5a, the force started to increase when the impactor contacted the fabric or the sample stage. In our impact testing device, the loadcell was placed under the sample stage. Therefore, the lower the force obtained, the better the protective performance of the fabric. Compared with the spacer fabric and the graphene-based aerogel/spacer fabric composite samples [30], the ecoflex/aerogel/spacer fabric composite samples exhibited a lower maximum impact force. For spacer fabric, the graphene-based aerogel/spacer fabric, and the ecoflex/aerogel/spacer fabric, the impact force was 95 N, 92 N, and 86 N, respectively, indicating better protective performance of the three-phase composites.

In addition, the force sensitivity of the ecoflex/aerogel/spacer fabric composite sensor in the impact process was also studied. As shown in Figure 5b, when the impact process was applied, the resistance of the sample increased sharply, and the peak value of ΔR/R_0_ reached 385%. When the ball bounced, the value of ΔR/R_0_ was stable at 21% and cannot return to 0. This is because its effective conductive path was damaged by the impact process although the network structure of the graphene-based aerogel is protected by ecoflex. Even so, the conductive network structure is less disrupted compared to graphene-based aerogel/spacer fabric composites [30].

In order to further study the impact stability of this composite sensor, the impact test was repeated 50 times, and we found that the anti-impact performance of the composite hardly diminished (Figure 5c). We calculated the protecting factor (PF) using the following formula: PF = (F_control_ − F_sample_)/F_control_ × 100. The lower PF value demonstrates better protective performance. It was found that the its protection factor only dropped from 60% to 55% (as shown in Figure 5d). Due to the unique structure of the spacer fabric and the cushioning performance of ecoflex, its anti-impact performance was improved. In order to further evaluate the stability of its sensing performance, its sensing performance of cyclic impact was tested. The impact and impact-sensing results are shown in Figure 5c and 5e, respectively. With the increase of impact times, the force peak value after 50 impacts increased from 86 N to 96 N, indicating that the protection performance weakened after 50 impacts, but the protection coefficient still reached 55%. Even after 50 impacts, the peak value of ΔR/R_0_ can still reach 330%, indicating its excellent impact sensing performance.

### 3.4. Temperature-Sensing Performance and Thermal Insulation Property of the Ecoflex/Aerogel/Spacer fabric Composites

**Figure 6:**
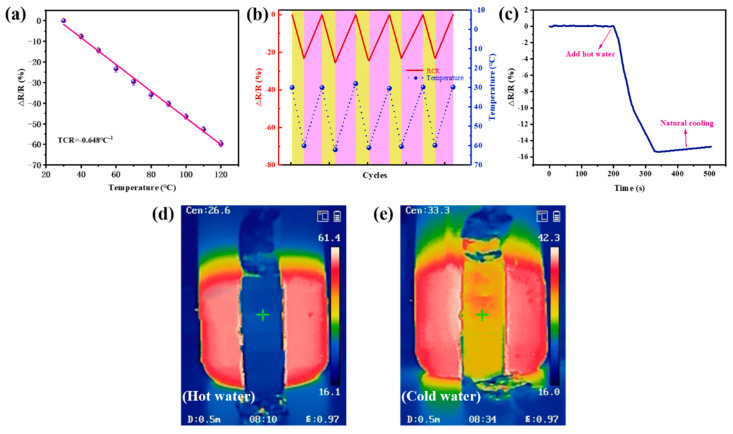
Thermosensitive performance and thermal insulation property of ecoflex/aerogel/spacer fabric temperature sensor. (**a**) Normalized relative resistance variation (ΔR/R (%)) of the sensor as a function of temperature (30–120 °C). (**b**) Reproducible temperature discrimination capacity of the sensor during the approach of cold and heat sources. (**c**) Detecting the temperature of the beaker before and after the addition of hot water. (**d**,**e**) IR thermal images of the beaker after the addition of hot water.

Temperature sensors have high requirements on the thermal sensitivity of materials, and ecoflex/aerogel/spacer fabrics have high thermal sensitivity, so it can be used as a temperature sensor. The sensitivity of evaluating the temperature response is the temperature coefficient of resistance (TCR), which is defined as TCR = (*ΔR*/*R*_0_)/*ΔT*, where *ΔR*/*R*_0_ is the resistance change rate and ΔT is the temperature change. Figure 6a shows that the resistance of the ecoflex/aerogel/spacer fabric decreases with increasing temperature, indicating a negative temperature coefficient (NTC) behavior of the composite sensor. There is a highly linear thermal response between 30 and 120 °C. A linear relationship is also important to simplify post-measurement processing steps.

The composite sensor exhibits NTC behavior, in which the resistance becomes smaller as the temperature increases, indicating that the intrinsic semiconductor behavior is dominated by thermally excited carriers [10]. This is because the process of reducing graphene oxide is limited by temperature and time, making it difficult to completely reduce graphene oxide. Therefore, the residual oxygen and hydrogen groups on the surface cause the graphene-based aerogel to potentially form a limited band gap semiconductor [40,41]. In addition, defective graphene can reduce the carrier density and thermal conductivity. If the carrier density is low, it will induce a better NTC effect. When the carrier density is very low, thermally excited carriers will resulting in greater resistance changes [10].

Five heating and cooling cycles were performed on the composite sensor at temperatures ranging from 30–60 °C, 60–90 °C, and 90–120 °C, while the resistance changes were recorded. As shown in Figure 6b and Figure S5 in the Supporting Information, the resistance changes significantly as the temperature increases or decreases, and the output signal remains consistent, indicating high repeatability and stability. Specifically, resistance decreases as temperature increases due to the negative temperature coefficient. When the temperature decreases through natural cooling, the resistance returns to its original state. It is important to note that it takes longer to recover resistance from higher temperatures than from lower temperatures because natural cooling takes longer.

To fully investigate its real-time thermosensitive response, we used the temperature sensor to detect the resistance variation produced by a beaker containing water at different temperatures (Figure 6c–e). The resistance decreased sharply when hot water was added, and increased slowly when it cooled naturally. The corresponding IR thermal images revealed that the composite also provides thermal insulation.

### 3.5. Thermal Insulation Property of the Ecoflex/Aerogel/Spacer Fabric Composites

**Figure 7:**
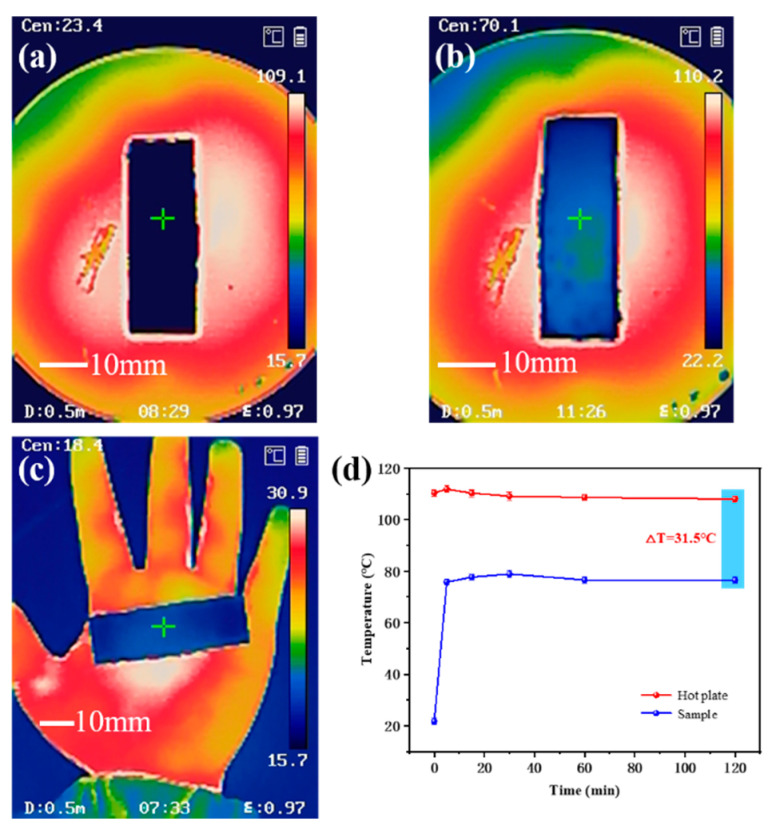
IR images of a ecoflex/aerogel/spacer fabric (thickness: 9 mm) (**a**) on top of a hot plate (110 °C), (**b**) on top of a hot plate (110 °C, 100 min), and (**c**) on a human palm. (**d**) IR shielding performances of the ecoflex/aerogel/spacer fabric. The sample size was 4.5 cm × 1.3 cm × 0.9 cm.

Aerogels are widely used for thermal insulation because of their low-density and highly porous internal structures [42]. Moreover, the upper and lower layers of spacer fabrics can trap air, and the ecoflex filled inside can also effectively insulate heat transfer, which provides good thermal insulation. Therefore, we investigated the thermal insulation performance of the ecoflex/aerogel/spacer fabric composite. By placing the ecoflex/aerogel/spacer fabric composite on top of a hot plate and a human palm, we were able to demonstrate that the composite was able to block IR transmission (Figure 7a–c). The heat transfer inhibiting effect of the composite was further demonstrated by placing it on a hot plate (110 °C). The 9 mm composite effectively blocked heat transfer; there was a 31.5 °C temperature difference between the opposite surfaces from the hot surfaces (Figure 7d). Heat penetration stabilized in the first 5 min, and the temperature subsequently remained almost constant over time. Even after 120 min, the temperature of the top surface of the composite remained at 76.5 °C, suggesting that heat was effectively blocked by the composite.

### 3.6. The Compression and Anti-Imapct Properties of the Ecoflex/Aerogel/Spacer Fabric Composites

To save the time of conducting an experiment, it is necessary to investigate the compression and impact behavior by numerical simulation. Based on the homogeneity assumption, a hyperelastic constitutive model of ecoflex/aerogel/spacer fabric composites was established. The deformation energy was derived from matrix deformation and fiber (spacer fiber) compression. The correctness of the constitutive relationship of composite is verified by comparing the finite element output results with the actual experiment. This provides a theoretical basis for the subsequent design and optimization of three-dimensional materials.

**Figure 8:**
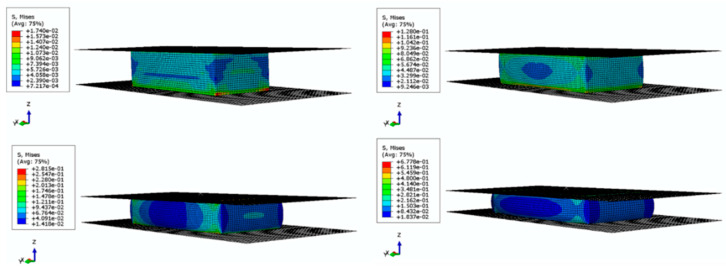
Von Mises stress plots of FE model in compression process.

The Von Mises stress plots of the composite during compression are shown in Figure 8.

**Figure 9:**
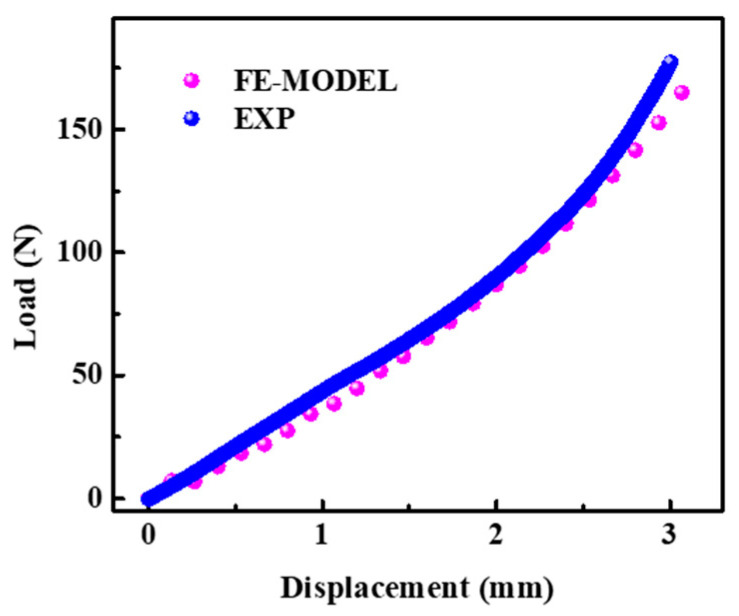
Load-displacement curves of compression.

The compression load-displacement curve of the composite is shown in Figure 9. It can be seen that the compression load-displacement curve of finite element simulation is in good agreement with the experimental results. With the increase of compression displacement, the load gradually increases and the increase rate becomes faster, indicating that the modulus gradually increases. The overall variation trend of load displacement is relatively consistent between the finite model analysis and experiment results. When the displacement is 3.0 mm, the peak value obtained by compression experiment is 177.2 N, and the peak value obtained by finite element simulation is 164.3 N. The peak error is 7.3% (less than 10%), which further demonstrates that the model can effectively simulate the compression behavior of ecoflex/aerogel/spacer fabric composites.

**Figure 10:**
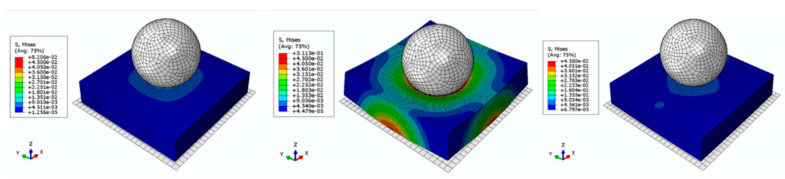
Von Mises stress plots of FE model in impact process.

The Von Mises stress plots of the composite during impact is shown in Figure 10. It can be seen that when the impact displacement reaches the maximum value, the stress between the impact ball and the sample reaches the maximum value. With the removal of the impact ball, the stress decreases.

**Figure 11:**
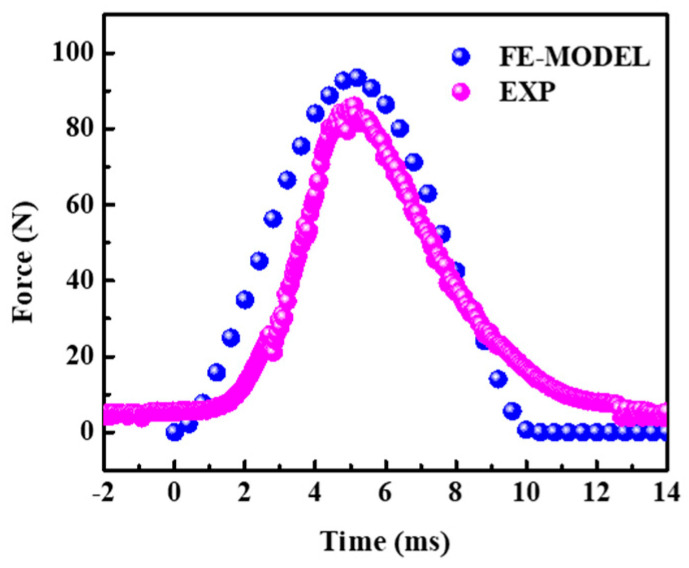
Load-time curves of impact test.

The impact load-time curve of composite is shown in Figure 11. It can be seen that the impact load–time curve of finite element simulation is in good agreement with the experiment results. The impact time obtained by the experiment is about 12.1 ms, and the peak value is 86.2 N. Meanwhile, the impact time obtained by finite element simulation is 10.2 ms, and the peak value is 94.1 N, correspondingly. The peak error is about 9.3%, less than 10%, indicating that the model can also effectively simulate the low-speed impact behavior of ecoflex/aerogel/spacer fabric composites.

## 4. Conclusions

In this work, flexible ecoflex/aerogel/spacer fabric composites with pressure sensing, impact resistance, temperature sensing, and thermal insulation properties were prepared. The as-prepared composite sensor exhibited great compressive strain sensing performance (strain range 0~55%, sensitivity 17.71 at strains of 40~55%; pressure range 0~335 kPa, sensitivity 0.125 kPa^−1^ at pressures of 0~15kPa) and cyclic stability. Meanwhile, the composite was able to respond to dynamic impacts, showing 55% of its protection factor and stable impact-sensing performance. The composite also exhibits a linear response to temperature, with a temperature coefficient of resistance (TCR) of −0.648 °C^−1^. In addition, the composite also shows great thermal insulation properties. After 120 min, the temperature difference between the material surface and the heating plate (110 °C) was maintained at 31.5 °C. Therefore, the composite can be used as a strain/pressure/temperature sensor with impact resistance and thermal insulation properties. The compression and impact process results of finite element numerical simulation analysis are in good agreement with the experimental results, which has certain guiding significance for subsequent simulation and optimization research.

## Figures and Tables

**Table 1:** Comparison of sensing performance with other sensors.

Materials	Sensitivity	Work Range	Response Time	Reference
Flat knitting spacer fabric/Conductive cloth tape	0.009 kPa^−1^	0~100 kPa	150 ms	[35]
Mxene	GF = 6.02	0~20%	None	[36]
TFEA/AAm	0.0059 kPa^−1^ (1~10 kPa), 0.0026 kPa^−1^ (10~100 kPa)	1~100 kPa	200 ms	[37]
Conductive cloth tape/Porous PDMS	0.023 kPa^−1^	0~200 kPa	155 ms	[38]
Electrode plates/Porous PDMS	0.0107 kPa^−1^	0~12 kPa	None	[39]
**Graphene**	**GF = 17.71 (0%~55%)** **0.125 kPa^−1^ (0~15 kPa)**	**0~55%** **0~335 kPa**	**92 ms**	**This work**

## Data Availability

The data presented in this study are available on request from the corresponding author due to privacy.

## Supplementary Materials

The following supporting information can be downloaded at: https://www.mdpi.com/article/10.3390/polym16111544/s1, Figure S1. Size of (a) compression samples and (b) impact samples of ecoflex/aerogel/spacer fabric. Figure S2. Schematic diagram of the impact experiment. Figure S3. Models of compression and impact sample. Figure S4. Schematics of compression and impact model. Figure S5: Reproducible temperature-discrimination capacity of the sensor during the approach of cold and heat sources. (a) Temperature range of 60 °C to 90 °C, (b) Temperature range of 90 °C to 120 °C.
