# Supplementary material for: Flexible and Multifunctional Composites with Highly Strain Sensing and Impact Resistance Properties
Source: Polymers (Basel). 2024 May 30;16(11):1544. doi: 10.3390/polym16111544 (PMC11174733; doi:10.3390/polym16111544)
Supplement: Supplementary file 1 [file polymers-16-01544-s001.zip › polymers-2976579-SI.pdf]

Supporting Information

Flexible and Multifunctional Composites with Highly Strain Sensing and Impact Resistance Properties

Shu Wang[1,2,†*], Jianyu Pu[1,†], Shuquan Xu[3,*], Yuanhao Tian[3], Qian Shu[3], Rui Zou[4] and Tonghua Zhang[1,*]

1 State Key Laboratory of Resource Insects, College of Sericulture, Textile and Biomass Sciences, Southwest University, Chongqing 400715, China; p836149527@email.swu.edu.cn (J.P.) ; Tel: +86-23-68251717; Fax: +86-23-68251228

2 College of Aerospace Engineering, Chongqing University, 174 Shazheng St, Shapingba District, Chongqing 400044, China

3 Southwest Technology and Engineering Research Institute, Chongqing 400039, China; yuanhao_tian@foxmail.com (Y.T.); 17723898496@163.com (Q.S.)

4 School of Materials Science and Engineering, Chongqing Jiaotong University, Chongqing 400074, China; ruizou@cqjtu.edu.cn (R.Z.)

* Correspondence: wangshu@swu.edu.cn (S.W.); xu_shuquan@126.com (S.X.); zhtonghua@aliyun.com (T.Z.)

Experimental section

Finite element analysis of the Ecoflex/Aerogel/Spacer fabric sensor

The size of the compression test is 45 mm×15.0 mm×10.0 mm, and the size of the impact test is 45.0 mm×45.0 mm×10.0 mm. The quasi-static axial compression test was carried out at room temperature using an EZ-L universal testing machine at a compression speed of 10 mm min^{-1}.

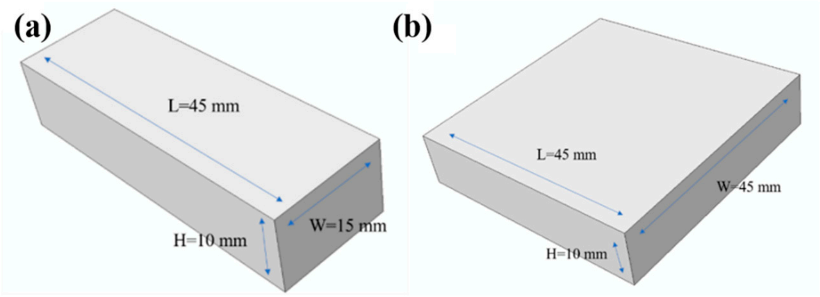

Figure S1. Size of a) compression samples and b) impact samples of ecoflex/aerogel/spacer fabric

The dynamic impact mechanical performance test is carried out with a ball impact test device, which mainly includes a small ball, a sample stage, a mechanical sensor, and a height control rod (as shown in Figure S2). The specimen is placed on the sample stage, and the specimen with specific size is shown in Figure S1(b). A ball (with a mass of 115.04 g) is placed at a fixed height

of 25.0 cm. When the ball touches the surface of the sample, the sensor get the corresponding signal. The impact velocity is calculated to be 2236 mm s^{-1}.

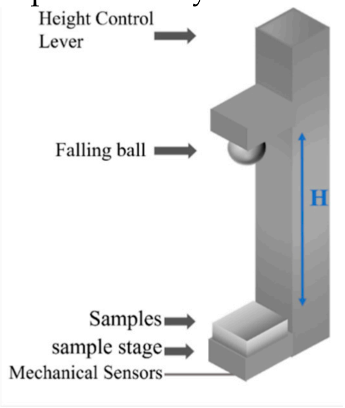

Figure S2. Schematic diagram of the impact experiment

Material model and constitutive model

After discarding the I2-related items on the basis of Rivlin, Yeoh retained the first three items to obtain a reduced polynomial constitutive model[1, 2]. In the case of large strains, the calculation results of this model are consistent with the experimental results. The specific form is as follows:

$$W = C_{10}(I_1 - 3) + C_{20}(I_1 - 3)^2 + C_{30}(I_1 - 3)^3 \quad \text{(S1)}$$

The Yeoh model can accurately predict uniaxial and planar tensile experiments. Therefore for compression of matrix Yeoh model is used.

In this work, the aerogel/ecoflex matrix is assumed to be a simple isotropic hyperelastic material, and the spacer fabric is assumed to be a transversely isotropic material. Assuming that the composite is a fiber-reinforced composite material, for the hyperelastic constitutive of this composite, a macroscopic hyperelastic constitutive model is adopted. In the established constitutive model, it is assumed that the fibers are uniformly distributed in the aerogel/ecoflex matrix in the direction of reinforcement, and the perfect combination between the matrix and the fibers does not occur slippage.

Assume that the strain energy function W is a scalar function[3] of the right Cauchy-Green strain tensor $\mathbf{C} = \mathbf{F}^T\mathbf{F}$ and the initial fiber orientation vector $\mathbf{a}_0$ i.e., $W = W(\mathbf{C}, \mathbf{a}_0)$, where F is the deformation gradient tensor. The elastic response of the composite is assumed to come from the matrix and fibers. The corresponding strain energy function W can be divided into two parts:

$$W = W(\mathbf{C}, \mathbf{a}_0) = W^M + W^F \quad \text{(S2)}$$

In Equation (S2), W^M refers to the strain energy from the matrix and W^F

refers to the strain energy from fiber compression.

The strain energy function of the fiber can be expressed by the right Cauchy-Green strain tensor invariant:

W（**C**, **a**0）=W(I_1, I_2, I_3, I_4)　　（S3）

a_0 refers to the fiber initial orientation.

The strain tensor invariants are: $I_1 = \mathrm{tr}\boldsymbol{C}$; $I_2 = \frac{1}{2}[(tr\boldsymbol{C})^2 - tr\boldsymbol{C}^2]$; $I_3 = \mathrm{det}\boldsymbol{C}$; $I_4 = \boldsymbol{a_0} \cdot \boldsymbol{C} \cdot \boldsymbol{a_0} = \lambda_a^2$

The strain energy function of the fiber section comes from fiber compression. The formula that defines a simple nonlinear model W^F is as follows:

$$W^F = \begin{cases} C_2(1-I_4)^2 + C_3(1-I_4)^4 & I_4 < 1 \\ 0 & I_4 \geq 1 \end{cases} \qquad \text{(S4)}$$

According to the constitutive model of the composite material constructed in this paper, the compression and impact finite element models (as shown in Figure S3) were respectively established, and the mechanical properties of the composite material were studied by the finite element method.

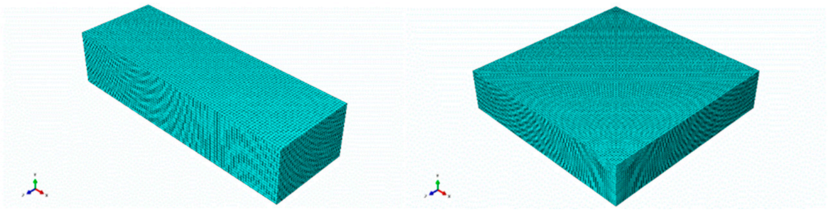

Figure S3. Models of compression and impact sample

The compression model is shown in Figure S4a. The matrix and fibers are simplified as a homogeneous composite material. The length, width and height are: L×W×H, L=45.0 mm, W=15.0 mm, H=10.0 mm. Discrete rigid bodies were established on the upper and lower surfaces of the homogenized composite material, with a length and width of 50.0 mm and 20.0 mm, respectively. The lower plate is used to simulate the sample platform to constrain the axial displacement of the sample, and the upper plate is used to simulate the pressure plate to compress the sample. The quasi-static compression time was set to 0.02 s. The compression displacement is 3.0 mm along the negative direction of the y-axis.

The impact model is shown in Figure S4b. The matrix and fibers are also

simplified as homogeneous composite materials, whose length, width and height are: L×W×H, L=45.0 mm, W=45.0 mm, H=10.0 mm. The metal plate and ball are respectively established as a three-dimensional discrete rigid body ball and a two-dimensional discrete rigid body. The diameter of the ball is 25.0 mm. The length and width of the two-dimensional discrete rigid body are both 50.0 mm and 50.0 mm. During the simulation, the ball and the sample were in direct contact, but an initial impact velocity was given to the ball based on the actual height until the ball was bounced away from the sample to complete a complete impact. The initial impact velocity is 2236 mm s^{-1} along the negative z-axis.

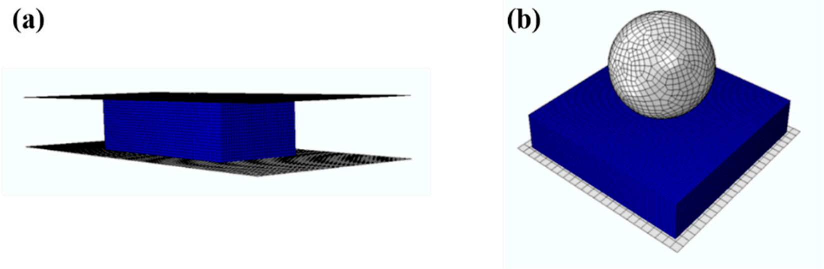

Figure S4. Schematics of compression and impact model

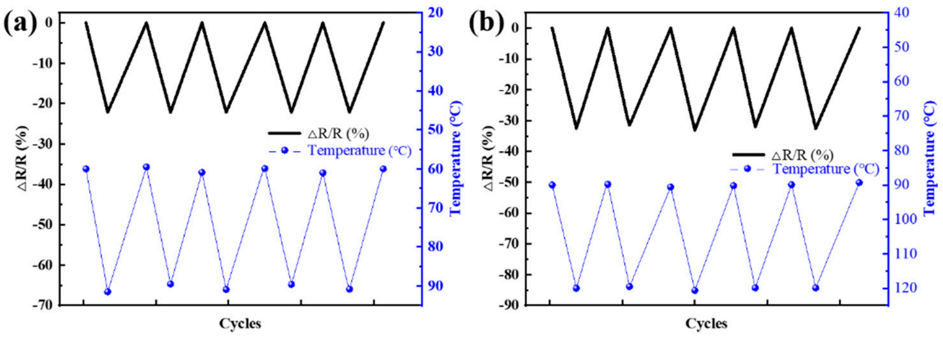

Figure S5. Reproducible temperature-discrimination capacity of the sensor during the approach of cold and heat sources. (a) Temperature range of 60℃ to 90℃, (b) Temperature range of 90℃ to 120℃.
